# The Variable Nature of Vitamin C—Does It Help When Dealing with Coronavirus?

**DOI:** 10.3390/antiox11071247

**Published:** 2022-06-24

**Authors:** Katarzyna Grudlewska-Buda, Natalia Wiktorczyk-Kapischke, Anna Budzyńska, Joanna Kwiecińska-Piróg, Jana Przekwas, Agnieszka Kijewska, Dominika Sabiniarz, Eugenia Gospodarek-Komkowska, Krzysztof Skowron

**Affiliations:** 1 Department of Microbiology, Ludwik Rydygier Collegium Medicum, Nicolaus Copernicus University in Toruń, 85-094 Bydgoszcz, Poland; katinkag@gazeta.pl (K.G.-B.); natalia12127@gmail.com (N.W.-K.); an.budzynska@wp.pl (A.B.); j.kwiecinska-pirog@cm.umk.pl (J.K.-P.); jana.przekwas@gmail.com (J.P.); gospodareke@cm.um.pl (E.G.-K.); 2 Department of Immunobiology and Environmental Biology, Institute of Maritime and Tropical Medicine, Medical University of Gdansk, 80-211 Gdansk, Poland; agnieszka.p.kijewska@gumed.edu.pl; 3 Antoni Jurasz University Hospital No. 1, 85-094 Bydgoszcz, Poland; dominika.sabiniarz@gmail.com

**Keywords:** SARS-CoV-2, COVID-19, ascorbic acid, natural antioxidants, inflammatory process, respiratory distress syndrome

## Abstract

Severe acute respiratory syndrome coronavirus 2 (SARS-CoV-2) is still spreading worldwide. For this reason, new treatment methods are constantly being researched. Consequently, new and already-known preparations are being investigated to potentially reduce the severe course of coronavirus disease 2019 (COVID-19). SARS-CoV-2 infection induces the production of pro-inflammatory cytokines and acute serum biomarkers in the host organism. In addition to antiviral drugs, there are other substances being used in the treatment of COVID-19, e.g., those with antioxidant properties, such as vitamin C (VC). Exciting aspects of the use of VC in antiviral therapy are its antioxidant and pro-oxidative abilities. In this review, we summarized both the positive effects of using VC in treating infections caused by SARS-CoV-2 in the light of the available research. We have tried to answer the question as to whether the use of high doses of VC brings the expected benefits in the treatment of COVID-19 and whether such treatment is the correct therapeutic choice. Each case requires individual assessment to determine whether the positives outweigh the negatives, especially in the light of populational studies concerning the genetic differentiation of genes encoding the solute carriers responsible forVC adsorption. Few data are available on the influence of VC on the course of SARS-CoV-2 infection. Deducing from already-published data, high-dose intravenous vitamin C (HDIVC) does not significantly lower the mortality or length of hospitalization. However, some data prove, among other things, its impact on the serum levels of inflammatory markers. Finally, the non-positive effect of VC administration is mainly neutral, but the negative effect is that it can result in urinary stones or nephropathies.

## 1. Introduction

The struggle with the coronavirus disease 2019 (COVID-19) pandemic caused by severe acute respiratory syndrome coronavirus 2 (SARS-CoV-2) has been ongoing since December 2019 [1], and the emergence of new SARS-CoV-2 variants is not conducive to ending the pandemic state. The number of COVID-19 patients continues to increase, and mortality, despite vaccination, remains high [2]. The new variants of SARS-CoV-2 represent a significant challenge in fighting the disease and reducing the spread of infection, despite current COVID-19 vaccinations. The latest variants of SARS-CoV-2 represent a significant challenge in fighting the disease and reducing the spread of infection, despite current COVID-19 vaccinations. According to the CDC, as of 12–22 May 2022, the variants of concern are delta (B.1.617.2) and omicron (BA.1, BA.2, BA.4, and BA.5) [3].

The aggravation of disease symptoms in the form of severe pneumonia contributes to acute respiratory distress syndrome (ARDS), cytokine release syndrome, and lymphopenia [4,5]. ARDS is associated with the oxidative stress generated during infection, which results from the production of cytokines and free radicals. The uncontrolled and unregulated secretion of inflammatory and pro-inflammatory cytokines is positively associated with the severity of viral infection and mortality [6]. The secretion of various pro-inflammatory cytokines such as TNF-α, IL-1β, and IL-6 leads to a hyperinflammatory response by recruiting macrophages, as well as T and B lymphocytes, in alveolar cells [7,8,9,10]. This process results in multi-organ failure, which can lead to death. People with reduced immunity are the most vulnerable to severe infection. These patients have low levels of inflammatory cytokines or chemokines such as IL-6 and TNF [10]. Currently, substances are being sought that would limit the effects of infection by modulating the immune system to reduce the inflammatory response or activate the antiviral response. Vitamin C (VC) is considered to be an antioxidant and is believed to strengthen immune function. It is a safe and affordable essential nutrient. This review aims to summarize the currently available scientific data on the role of VC in supporting the treatment of COVID-19 patients and the possibility of alleviating symptoms of the disease.

## 2. The Course of the Inflammatory Process during SARS-CoV-2 Infection

Angiotensin converting enzyme 2 (ACE2) has been identified as the primary cell receptor for SARS-CoV-2 entry into host epithelial cells. The SARS-CoV-2 spike (S) glycoprotein binds to ACE2 via the receptor-binding domain (RBD) [11]. Upon receptor involvement, several host serine proteases, including TMPRSS2, TMPRSS4, furin, and endosomal cathepsins, cleave the SARS-CoV-2 S protein at the junction between the S1 and S2 fragments, allowing fusion of host and viral membranes and delivery of the viral genome to the cytosol. In addition to ACE2, many other host molecules reportedly support the binding of SARS-CoV-2 to cells and act as entry factors, including CD147, neuropilin-1, sialic acid, and heparan sulfate [12,13].

**Figure 1:**
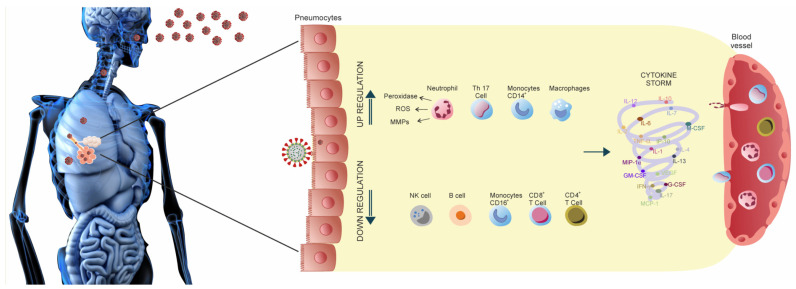
Cytokine storm in COVID-19.

Numerous studies suggest a significant role for several pro-inflammatory factors. These factors are interleukins (IL)-2, -6, -8, -10, -1β, tumor necrosis factors (TNF), interferons (IFNs), and granulocyte-macrophage colony-stimulating factor (GM-CSF) in the course of the disease COVID-19 [14,15,16]. In addition, chemokines, including CCL2, CCL3, CCL5, CXCL10, and other chemotactic cytokines [17], have been described as essential determinants of mortality during SARS-CoV-2 infection. Most of the co-expression of these genes has been well-described as actively expressed on the mucosal gene expression of antimicrobial peptides in inflammatory conditions caused by proinflammatory diseases, such as inflammatory bowel disease [18] or even cancer [19]. Their expression is also active in stereotyped and specific human responses to bacteria [20]. Interleukin-1β plays a pivotal role in the induction of cytokine storms due to uncontrolled immune responses in COVID-19 infections [21]. At the same time, IL-6 may even serve as an early biomarker for monitoring inflammatory and immune responses in COVID-19 [10]. In turn, the level of TNF-α is associated directly with the probability of hospitalization and severe COVID-19 [22]. High levels of TNF-α and other proinflammatory interleukins lead to ARDS aggravation and widespread tissue damage [23]. Interferon-inducible chemokines, such as CXCL10 (IP-10), are one of the leading players in the antiviral responses [24], while CXCL8 acts as a trafficking mediator for neutrophils [25]. SARS-CoV-2 infection is closely related to the cytokine storm. This storm is a highly deadly immune disorder characterized by the rapid proliferation and hyperactivation of NK cells, macrophages, and T lymphocytes, as well as the excessive secretion of over 150 chemical mediators and inflammatory cytokines by non-immune and immune cells [26]. This phenomenon plays a crucial role in the progression of SARS-CoV-2 infection and may be a significant cause of multiple organ damage and increased mortality in immunocompromised patients. IL, IFN, TNF, and GM-CSF are the main cytokines involved in generating cytokine storms in COVID-19 (Figure 1) [27]. Cytokines are generally divided into two categories based on their functionality during infection: pro-inflammatory cytokines/factors (IL-6, IL-12, IL-1β, IFN, and TNF) and anti-inflammatory cytokines/factors (IL-4, IL-7, IL-10, and TGF-β).

The release of multiple cytokines is associated with various clinical symptoms, such as the over-secretion of IFN-γ, which causes headaches, chills, dizziness, fatigue, and fever [28]. Similar to IFN-γ, TNF-α causes flu-like symptoms, along with fever, fatigue, and malaise, but it can also lead to lung damage, leaky blood vessels, heart failure, and acute-phase protein synthesis [29]. The secretion of IL-6 causes vascular leakage syndrome, activating clotting and complement pathways, leading to clear indications of cytokine release syndrome, i.e., blockage of small blood vessels. Moreover, IL-6 has been associated with the induction of cardiomyopathy by stimulating coronary disease and myocardial dysfunction [30]. Additionally, severe cytokine release syndrome can occur due to the activation of endothelial cells, and endothelial dysfunction can result in hypotension, capillary leakage, and impaired blood clotting. In summary, the inappropriate secretion of various cytokines leads to the activation of a cytokine storm, causing immunopathogenic damage to multiple organs and tissues, even in the presence of a strong, suppressive immune system response.

**Figure 2:**
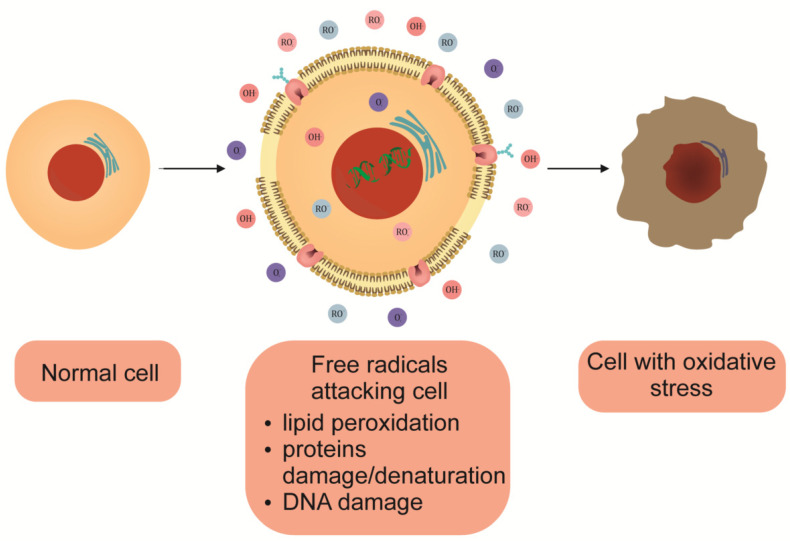
The effect of oxidative stress on the eukaryotic cell.

From the point of view of supporting the treatment of severe COVID-19 cases, it is essential to search for substances that will mitigate the effects of uncontrolled damage in the body and reduce the risk of death of patients. A crucial, vital element of the host immune response during viral infection is the production of reactive oxygen species (ROS) and reactive nitrogen species (RNS), which leads to the destruction of infected cells in the early stages of the immune response [31]. Despite the beneficial effect of producing these molecules in the early stages of infection, their excessive production leads to oxidative stress, an imbalance between pro- and anti-inflammatory cytokines, and damage to tissues and organs (Figure 2). Many anti-inflammatory substances can neutralize the production of free radicals. These include glutathione; carotenoids; polyphenols; vitamins, such as C, D, and E, selenium, and zinc.

## 3. Natural Antioxidants to Treat or Support COVID-19 Patients?

The course of COVID-19 can be mild, moderate, or severe. The treatment regimen depends on the patient’s condition. The World Health Organization (WHO) recommends treatment with casirivimab and imdevimab (for those with the highest risk of hospitalization), administering convalescent plasma for the treatment of patients with non-severe COVID-19, and using IL-6 receptor blockers (tocilizumab or sarilumab) [32]. However, the WHO advises against using hydroxychloroquine, chloroquine, or lopinavir/ritonavir to treat COVID-19 [32]. Patients with moderate-to-severe pneumonia may develop (life-threatening) sepsis, followed by septic shock, which is associated with multiple organ failure (MOF) and high mortality [33]. Septic shock during COVID-19 is characterized by the increased production of ROS and RNS [33]. SARS-CoV-2 affects the intracellular level of GSH (glutathione) by reducing intracellular functions [34]. Therefore, an alternative to supporting the treatment of COVID-19 patients may be antioxidant therapy, which has been known since Hippocrates, who used myrrh for anti-inflammatory, medicinal purposes [35].

The primary mechanism of antioxidants is to increase intracellular GSH levels and scavenge free radicals, thereby protecting DNA, cytosolic proteins, and membrane lipids [35]. GSH deficiency is associated with a more severe manifestation of COVID-19. Among 4 COVID-19 patients, Polonikov et al. [36] observed a decrease in ROS (in patients with higher GSH levels) and a shorter course of the disease. In contrast, lower GSH levels have been associated with more severe disease symptoms and higher ROS [36]. SARS-CoV-2 has been shown to affect intracellular GSH levels by reducing the function of intracellular NRF2, which plays a crucial role in protecting cells from oxidative damage by regulating GSH production [34]. Horowitz et al. [37] showed that GSH supplementation in 2 COVID-19 patients reduced dyspnea (within 1 h of GSH administration), and this improved with each subsequent dose. Hiedra et al. [38] administered 1 g of VC intravenously (every 8 h for 3 days) to 17 COVID-19 patients. These researchers demonstrated reduced mortality, decreased intubation and mechanical ventilation, a significant decrease in inflammatory markers (ferritin and D-dimer), and a tendency toward a reduced need for FiO_2_ [38]. In turn, Colunga Biancatelli et al. [39] recommended a combination of VC and quercetin (for a synergistic antimicrobial effect) in treating COVID-19 patients. Several studies on the impact of VC supplementation are summarized in Table 1.

Other compounds are also involved in antioxidant therapy. Antioxidant therapy may involve a combination of two or more compounds. A study of the effects of vitamin C, vitamin E, N-acetylcysteine (NAC), and pentoxifylline (Px) on 110 COVID-19 patients was conducted by Chavarría et al. [35] The researchers found that antioxidant therapy improved survival rates. The improvement was measured using the SOFA (sequential organ failure assessment) score, the Apache II (Acute Physiology and Chronic Health Evaluation) score, the SAPS II (Simplified Acute Physiology Score), the COVID-GRAM (critical illness prediction tool), and the GCS (Glasgow Coma Scale). In addition, the researchers also took into account the hospitalization for antioxidant therapy, the duration of mechanical ventilation, and the length of stay in the intensive care unit [35].

An example is the combination of NAC + Px in the early stage of the disease. This combination leads to a reduction in inflammation and cell damage [34]. In turn, Chavarría et al. [35] showed that combining Px supplementation with any antioxidant served to decrease IL-6 and CRP levels in COVID-19 patients. Serum zinc content (SZC) influences the progression of COVID-19 in the disease and may constitute a valuable, helpful biomarker [40]. The study data clearly show that many COVID-19 patients were zinc-deficient. More complications and deficiencies were associated with prolonged hospitalization and increased mortality [41].

Antioxidant therapy may be beneficial in those patients with comorbidities who take medications each day on a permanent basis. Intracellular GSH levels in RBCs are reduced in patients with type II diabetes [49]. Therefore, supplementation with antioxidants during COVID-19 may be helpful in this group of patients. Sinaci et al. [50] showed that the levels of vitamin D in pregnant women with moderate/severe courses of COVID-19 were lower (13.69 ng/mL) compared to those of pregnant women with a mild approach (13.69 ng/mL). In addition, Schmitt et al. [51] obtained similar results. They showed that the level of vitamin D in pregnant women (patients and controls) was <20 ng/mL, while in the case of mild COVID-19, the level of vitamin D in pregnant patients was lower (<12 ng/mL) [51].

In contrast, in another study, vitamin D levels were not associated with the level of severity of COVID-19 during pregnancy as similar levels of vitamin D deficiency were found in COVID-19-positive patients and controls [52]. Therefore, studies are ongoing, including clinical ones, on the use of antioxidants to treat COVID-19 patients [53]. A list of the most important antioxidants is presented in Table 2.

In addition to antioxidants, diet can be an essential consideration when COVID-19 is mild. Many ingredients of natural origin can influence the course of the disease. An example is propolis, considered a natural means of supporting the immune system. Propolis promotes the immunoregulation of pro-inflammatory cytokines, reducing IL-6, IL-1, TNF-α, Jak2/STAT3, and NF-κB. The reduction effects lower the risk of cytokine storm syndrome (Figure 1).

Phytochemicals that show promise for inhibiting human coronaviruses include quercetin, myricetin, and caffeic acid, which are all constituents of propolis [65]. Propolis is used to treat comorbidities, such as diabetes, cancer, and hypertension [66]. Flavonoids also have a positive effect on inhibiting the cytokine storm because they regulate inflammatory mediators; inhibit endothelial activation, NLRP3 inflammasome, Toll-like receptors (TLR), and bromodomain protein 4 (BRD4); and activate Nrf2 [67]. The sources of flavonoids are herbs, fruits, and vegetables. Su et al. [68] showed that baicalein and baicalin inhibited SARS-CoV-2. Additionally, baicalin is an ACE2 inhibitor [69]. Other compounds that inhibit SARS-CoV-2 replication include curcumin, quercetin, kaempferol, capsaicin, sesamine, cyanidins, puerarin, scutellarin, ursolic acid, rutin, cesalamin B, ebsalene, galangin analogues, ellagic acid, and coumarin [70,71,72,73,74]. On the other hand, activity against SARS-CoV-2, due to a high affinity with the S protein of RBD, has been demonstrated for hesperidin, curcumin, braziline, and galangin [75]. Another active ingredient in the food is allicin, the source of which is garlic. Allicin inhibits inflammation by inhibiting TNF-α, which induces the expression of IL-1β, IL-8, IP-10, and IFN-γ [76]. Thus, consuming garlic may be helpful during a mild course of COVID-19 [57].

Supplementation therapy with antioxidants may contribute to a decrease in mortality among COVID-19 patients. However, caution should be exercised as many oxidant-based treatments have produced negative or inconclusive results [77]. Additionally, data on the possible effects of VC on COVID-19 in children are not yet available.

## 4. Vitamin C

Ascorbic acid (AA), which is a saccharide derivative produced from D-glucose (by plants and animals) or D-galactose (by plants), was first isolated in 1928 from pepper and adrenal gland extracts [78]. The AA molecule has four stereoisomers: biologically active L(+)-ascorbic acid (VC), D-ascorbic acid and L-isoascorbic acid (inactive forms), and D(-)-isoascorbic acid (which has little biological activity).

VC began to be used in the early 20th century to treat scurvy [79]. The inability to synthesize VC in primates due to a mutation in the gene encoding the L-glucono-γ-lactone oxidase (Gulo) which is involved in the final step of AA biosynthesis makes it necessary to provide it via the dietary route. The enzyme which oxidizes L-gluconolactone to AA is also lacking in some animals—certain species of fish, birds, bats, and guinea pigs, which provides a useful animal model for research [80]. Nowadays, hypovitaminosis C leading to scurvy, although still reported, is rare because of the awareness of the need to supply it via an adequate diet. The Recommended Dietary Allowance (RDA) of VC is an average of 75 mg for women and 90 mg for men. (RDA values may vary depending on the country.) VC deficiency can result from malabsorption disorders, malnutrition, and improper diets that reduce AA absorption, and it is also observed in patients with infections or cancer, and in critically ill patients. Increased supplementation should apply to women with pregnancies (especially multiple pregnancies), lactating women, smokers, people with hypertension or diabetes, and those living under stress [81,82,83].

Ascorbate is transported into the cell across cell membranes by sodium-dependent VC transporters (SVCT1 and SVCT2). In contrast, in the transport of the oxidized form, dehydroascorbic acid (DHA), which is reduced again in the cell, glucose transporters (GLUT) are involved [84]. In healthy individuals, plasma VC concentrations are approximately 50–100 μM [85]. Accumulation of AA in organs and tissues varies, with the highest levels found in endocrine cells and neurons, mainly in the adrenal and pituitary glands [86]. The kidneys excrete excessive amounts of VC; therefore, in most cases, there is no danger associated with its consumption, even at a dose that exceeds its daily requirement (1 to 200 g) [87]. Possible adverse side effects of high doses of VC include abdominal bloating and/or transient osmotic diarrhea and polyuria. Controversial, however, is the increased risk of urinary stone formation due to the increased concentration of urinary oxalate under the influence of VC doses exceeding 1 g per day. Therefore, one may be mindful of excluding this supplementation in individuals with kidney stones or renal impairment [88,89,90].

Ascorbate is a cofactor for a family of biosynthetic and regulatory metalloenzymes, such as hydroxylases (prolyl 4-hydroxylase and lysyl hydroxylase), or oxygenases (copper-containing monooxygenases, iron (II)-, and 2-oxoglutarate-dependent dioxygenases). Consequently, ascorbate is involved in the stabilization of the tertiary structure of collagen, as well as in the synthesis of carnitine and hormones (noradrenaline/adrenaline and peptide hormones). For this reason, with VC deficiency, the integrity of basement membranes is weakened, and due to disorders in the synthesis of connective tissue, wound healing is impaired, and the risk of osteoporosis and fractures increases [91,92,93]. AA has also been shown to positively affect the efficiency and accuracy of epigenetic reprogramming by demethylating the lysine 36 of histone H3 (H3K36). This process facilitates the formation of induced pluripotent stem cells (iPSCs). AA also reduces the frequency of the unwanted spontaneous differentiation of iPSCs [94].

An essential role of AA, an electron donor, is the antioxidant activity mentioned earlier—protecting cells from free radicals, such as peroxide radicals, hydrogen peroxide, singlet oxygen, and hydroxyl radicals—formed during metabolism. Ascorbic acid, because of oxidation, converts to an ascorbic anion which, after donating an electron, becomes a nonreactive ascorbic radical. DHA, characterized by the same biological activity as the reduced form, is formed by losing another electron [84,95].

**Figure 3:**
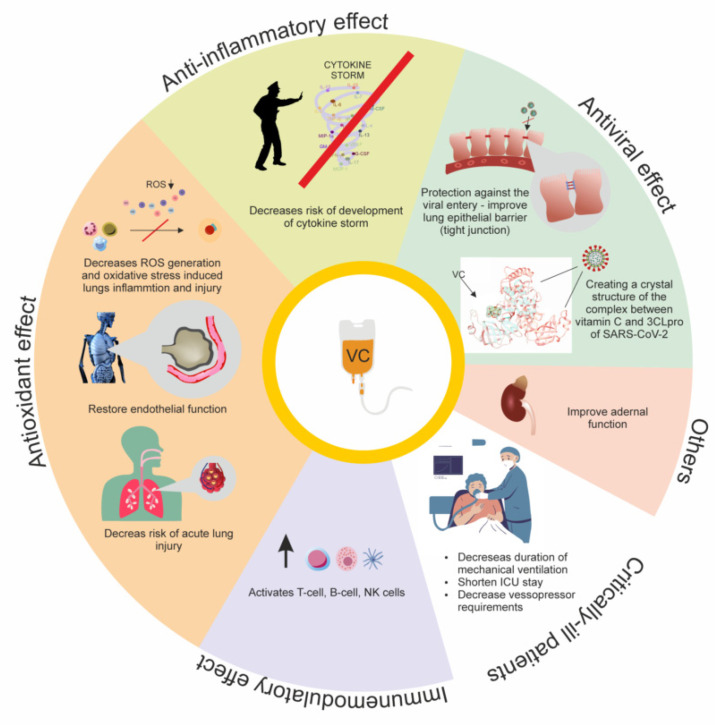
The effect of vitamin C in SARS-CoV-2 infection.

VC plays a vital role in the proper functioning of the immune system. Its concentration in immune cells (lymphocytes, monocytes, and neutrophils) in individuals consuming ≥100 mg ascorbate per day is up to 100 times higher than plasma concentrations [93,96]. AA affects immune cell function in the hypoxic environment in inflammation and cancer because it is required for the optimal hydroxylase activity that regulates the activity of the hypoxia-inducible factors (HIFs) that direct inflammatory and immune responses. The activation of HIFs prolongs neutrophil survival and their antibacterial function. HIFs may also be important in T-cell differentiation, activation, and function [97]. Thus, AA affects lymphocyte development and function. Studies have shown that AA plays a role in the normal maturation of T cells in the thymus, probably through the epigenetic modulation of gene expression. Its involvement in regulating the activity of mature T lymphocytes is also considered [98]. In the case of B lymphocytes, results remain inconclusive in establishing the effect of VC on their proliferation and function [99]. VC, on the other hand, may protect neutrophils from the ROS that they produce and from oxidative damage, as well as increasing the motility and migration of various peripheral blood leukocytes, such as lymphocytes or polymorphonuclear leukocytes, in innate immune responses (Figure 3) [100].

The effect of VC on the immune system has been observed in vivo studies. With age, there is excessive apoptosis and a gradual decrease in the proportion of functional T cells. In older adults, after 3 months of supplementation (500 mg/day), improvements in the immune functions—increased lymphocyte proliferation and interleukin-2 release—were found [101]. In turn, in the studies by Bozonet et al. [102], an increase in VC levels in the neutrophils of individuals with a low VC intake, as influenced by administration in its natural form (kiwi fruits), resulted in a significant increase in neutrophil chemotaxis and superoxide generation. The role of antioxidants in altering the expression of adhesion molecules and inhibiting monocyte adhesion to endothelial cells (ECs) in blood vessels observed in early atherosclerotic lesions has also been suggested [103]. This conclusion is supported by the study of Woollard et al. [104], which demonstrated the normalization of monocyte adhesion under the influence of VC supplementation in people showing reduced VC levels. It is difficult to assess the role of AA in NK cells. Studies indicate that it may have a positive effect by increasing the cytotoxicity of impaired NK cells, while no AA effect on properly functioning NK cells has been observed [99,105,106].

Brain cells, particularly susceptible to oxidative damage, show increased sensitivity to VC deficiency. Due to the synaptic activity and intense neuronal oxygen metabolism of central nervous system cells and the associated increased production of free radicals, a sufficiently high level of antioxidants is required. In the case of glial cells, such an oxidant is glutathione, and in nerve cells, it is mainly AA [107,108]. Thus, there is a possible link between ascorbate deficiency and neurodegenerative diseases, including Alzheimer, Parkinson, and Huntington disease, as well multiple sclerosis [109].

Currently, much research is focused on using VC as a supplement in treating many diseases (such as cancer) and infections, including sepsis and septic shock [110]. In the case of cancer, attention has been drawn to the contribution of VC in reducing the toxicity of chemotherapy. The positive effect of using VC in preventing the development of malignant tumors, such as those caused by colorectal adenocarcinoma, lung cancer, and endometrial cancer, has also been described. Moreover, its synergistic effect with many chemotherapeutic agents (including carboplatin, arsenic trioxide, and paclitaxel) used in the treatment of various cancers has been indicated [87,111,112,113]. This effect is probably related to the fact that VC, in addition to its antioxidant properties, may exhibit pro-oxidant effects. If catalytic metals, especially iron and copper, are present in the environment, AA reduces them (Fe^3+^ → Fe^2+^, Cu^2+^ → Cu^+^). Their new forms can react with oxygen, forming superoxide or hydroxyl radicals [84,114]. The effect of AA cytotoxicity on inhibiting the growth rate of many cancer cell types was demonstrated in vitro and in vivo studies by Chen et al. [115]. Parenteral administration of ascorbate in mice bearing glioblastoma xenografts at pharmacological doses (>0.2 mM) resulted in sustained ascorbate radical and hydrogen peroxide formation in the extracellular space, followed by the autophagy of cancer cells, while having no toxic effects on normal tissues. The process is associated with the inhibited activity of enzymes that neutralize oxidative stress in cancer cells, leading to a reduced ability to metabolize H_2_O_2_ compared to normal cells [116]. Elevated free-radical levels can lead to the depletion of antioxidant mechanisms and the death of cancer cells. Apoptosis of breast cancer cells induced by AA at physiological concentrations (100 µM) was observed by Hong et al. [117], while Tronci et al. [118] demonstrated the antitumor effect of VC against papillary thyroid carcinoma cells. The oral intake of VC by patients with malignant tumors likely has no impact because, at lower concentrations, VC exhibits antioxidant, rather than pro-oxidant, activity [119]. However, more clinical studies are needed to confirm the positive effects of VC in oncology patients. One should also consider the results of research conducted on leukemia and lymphoma cell lines by Heaney et al. [120], which indicated that VC could inhibit the effects of anticancer drugs, such as doxorubicin, which produce reactive oxygen species.

Since VC can affect the cytokines secreted by immune cells, its beneficial effects have been shown in many studies using animals infected with viral and bacterial infections or *Candida albicans*. In studies, decreased or increased production of the pro-inflammatory cytokines TNF-α, IFN-γ, IL-6, and IL-1β, and increased production of anti-inflammatory IL-10 and IL-4, depending on the cell type, as well as inflammation stimulating factor, have been observed under its influence [121,122,123,124,125]. VC deficiency increases the risk of infection, which is why pneumonia was one of the most common complications of scurvy found in the 20th century [126]. At the same time, due to an overactive inflammatory response resulting in increased metabolism, increased demand for VC is observed in the course of infection. This demand increases significantly when the disease is more severe [127]. A study by Fisher et al. [128] found that, in mice treated with lethal doses of lipopolysaccharide, parenteral administration of AA attenuated proinflammatory states and reduced microvascular thrombosis. The attenuation prevented pulmonary vascular damage in the course of sepsis. Another paper [129], which demonstrated in a mouse model the enhancement of the alveolar–bronchial epithelial barrier function, confirms the potency of VC in preventing multiple organ dysfunction syndromes and treating sepsis. However, the results of clinical trials are not as conclusive. Shrestha et al. [130] described that using VC improved critically ill patients and significantly reduced their intensive care unit (ICU) stay. Hemilä and Chalker [131] reached similar conclusions with a meta-analysis that evaluated the effect of VC on the length of stay and the duration of mechanical ventilation of patients in the ICU. Fowler et al. [132], assessing the impact of 96-h intravenous VC infusion in patients with sepsis and acute respiratory distress syndrome, found no reduction in vascular damage, changes in organ failure, or C-reactive protein levels. However, there was a significant reduction in 28-day patient mortality compared with the placebo group. In contrast, different results (no improvement in mortality) were shown in patients with severe sepsis or septic shock in the following two studies [133,134]. Therefore, further randomized controlled trials are needed to evaluate the role of VC in the treatment of patients with sepsis.

As previously mentioned, it has also been suggested that VC may be used in the treatment of various viral infections. A daily dose of 1 g of VC is widely believed to reduce the risk of respiratory tract infections. However, not much research supports this assumption [135,136,137,138]. In contrast, supplementation at a dose of 0.25–1.0 g/day is recommended for athletes undergoing intensive training and/or during periods of increased risk of upper respiratory tract infections so as to reduce the incidence or shorten the duration of the common cold [139,140]. VC probably does not have a direct virucidal effect, and the impact of reducing viral loads in infected cells is due to its immunomodulatory properties, as described previously [141]. In a study using Gulo knockout (−/−) mice as the study model, it was found that their infection with the H3N2 or H1N1 influenza viruses resulted in more significant lung damage and higher mortality compared to wild-type mice. The effect was associated with an inadequate immune response, including reduced IFN-α/β production in VC-deficient mice [142,143].

During infection, an oxidative imbalance in the host cells occurs. The imbalance results in the overproduction of cytokines and contributes to a severe inflammatory response which, in respiratory infections, leads to lung tissue damage [144]. Furthermore, viruses exploit changes in redox homeostasis for their replication by activating various cellular pathways. VC, through its properties, reduces the oxidative stress that occurs during infection and inflammation due to the activation of phagocytes and ROS release. The VC-mediated reduction of ROS prevents ROS-induced lung damage in the course of viral infections [125,145]. Increased survival associated with AA treatment was observed by Valero et al. [146] in mice infected with the Venezuelan equine encephalitis virus and by Cai et al. [147] in restraint-stressed mice infected with the H1N1 virus.

In contrast, a study conducted on a group of patients with severe viral pneumonia with respiratory failure did not show a reduction in their mortality from receiving VC as compared to a control group [148]. In contrast, the evaluation of the effect of VC on the treatment of patients with herpes zoster has shown a reduction in the incidence of postherpetic neuralgia, as the antioxidant activity may play an essential role in reducing the pain caused by herpes zoster [149]. However, due to the limitations of the research, including the lack of the measurement of plasma VC concentration and the heterogeneity of the groups compared, the authors suggest conducting a multicenter, randomized study.

### 4.1. Variability of Genes and Their Expression Related to VC Metabolism

#### 4.1.1. Genetic Background of VC Carriers and Related Genes

It could be noticed by tracking the interactions described in the scientific literature dedicated to VC by web interface for generating hypotheses about gene function, analyzing gene lists, and prioritizing genes for functional assays [150] and these VC carriers (Solute Carrier Family 23 members—sodium-dependent SLC23a genes), most of the genes described as involved in a response to COVID-19 were noticed in the mucosal gene expression of antimicrobial peptides in bowel disease [18], the expression of genes engaged in liver cancers [151], and in drug-induced liver injuries [152]. These genes were also involved in expression patterns in primary human liver tissue connected to diabetes, drug response, lipid levels, prostate cancer [153], tumor gene expression [154], a drug-cell interaction in multiple myeloma patients [155], and oncogenic pathway signatures [19], but also in a pluripotent state of stem cells [156], genes characteristic for central nervous system distinct sites [157], and, finally, distinctive gene expression patterns in human mammary epithelial cells and breast cancers [158].

A list of genes involved in the above interactions, and related to the solute carrier family 23 (*slc23a1*, *a2*, and *a3*) and solute carrier family 2 (*slc2A1* and *A3*) were input into the ConsensusPathDB [159] and analyzed for grouping into pathways related to COVID-19. This procedure enriched transcription factor target sets with calculated *p*-values and q-values. Then, all statistically significant, selected groups were rechecked for any relation to the VC data in scientific literature. Surprisingly the presence of the slc23 subfamily and/or slc2 subfamily was annotated in the miRNA sets, miR-223-3p, miR-203a-3p, and miR-122b-5p. The miR-223-3p set was mentioned as being involved in regulating the SARS-CoV-2-induced inflammatory pathology [160]. According to the authors, the negative fold of the expression of miR-223-3p suggested contribution in vivo to limiting the excessive lung inflammation induced by SARS-CoV infection, and the inhibition of the miRNA-223 increases the mRNA expression levels of NLRP3 inflammasome and pro-inflammatory chemokines CXCL10, CXCL2, and IL-1ß. In this set, only *slc2a4* (*glut4*) was present. Still, according to Shaghagi et al. [161], it is postulated that VC accumulation in cells occurs partly through DHA transport by the carriers of the SLC2A family. Another set, miR-122b-5p, is much more interesting, despite its low *p*-value (*p* < 0.01). That set of miRNAs differed depending on whether study participants were healthy or had moderate-to-severe COVID-19. This differentiation suggests that the presence of long and short RNAs in human cells is integral to the post-transcriptional gene regulation of the gene set involved in the response to infection and the relevant to RNA of SARS-CoV-2.

Polymorphisms and the pathological relevance of genes *slc23a1* and *slc23a2* were examined for population differences in population differentiation by Eck et al. [162], and the authors found differences between alleles of 2 analyzed genes, depending on the origins of the sample donors: self-described African Americans differed from people self-described as Caucasian. A genetic variant (rs33972313 C/A/G/T) was found in exon 8 of the SLC23A1 locus. It is suggested that this missense causes a conformational change or protein failure responsible for lower concentrations of circulating ascorbic acid (−5.98 µmol/L) in the general population [163]. Moreover, Zanon-Moreno et al. [164] found that the presence/absence of rs1279386 (A>G) SNP is related to the different risks of primary open-angle glaucoma. They also demonstrated that the SLC23A2 genotype modifies the serum levels of VC [164].

A similar problem concerns *slc2a1* gene transporting, with the exception of glucose and dehydroascorbic acid. The polymorphism of that gene, especially SNP rs1385129 (A/G), is known to be related to diabetic cardiovascular disease (CVD) with diabetes mellitus type 2 (T2DM) [165] and diabetic microvascular complications [166,167]. In turn, the *slc2a2* gene’s polymorphisms (rs5438, rs8192675) may play a role in influencing high-density lipoproteins, and thus the metabolic risk of cardiovascular disease [168] in European Americans. Moreover, the decreased expression of GLUT1 (*slc2A1*) is critically involved in the disease progression of SARS-CoV-2 infection [169].

Another gene, *ace2* also known to be polymorphic, did not exhibit a statistically significant effect on COVID-19. According to Ivanov et al. [170], who performed experiments in vitro on SAEC and HMEC cell cultures, it is possible that AA can directly modulate the expression of the *ace2* gene and indirectly modulate individual susceptibility to COVID-19. This fact can support the assumption about the crucial role of VC in preventing infection and limiting the multiplication of the virus in the early stages of the disease.

#### 4.1.2. Absorption of VC and the Microbiome

Not only genetic variation can affect the level of VC in organisms. Heskett et al. [171] proved that the presence of enteropathogenic *E. coli* (EPEC) in gut microbiota could affect the uptake of ascorbic acid via the dysregulation of its transporters’ (SVCT1 and SVCT2 encoded by the *slc23a2* and *slc23a1* genes) expression.

Among many factors affecting the absorption of VC in the gastrointestinal tract, the intestinal microbiome is one which has the most influence. Commensal microorganisms affect both the synthesis of specific vitamins and the modulation of their absorption [172,173]. Subramanian et al. investigated the effects of bacterially derived lipopolysaccharide (LPS) on AA homeostasis in enterocytes. They showed that treating cell lines (Caco-2 line) affects the reduction of SVCT1 and SVCT2 proteins, mRNA, and hnRNA expression. The reduction of the expression of these elements, in turn, inhibits the absorption of ascorbic acid. Downregulating the expression of ascorbic acid transporters and the transcriptional regulation of SLC23A1 and SLC23A2 genes were the main reason for the inhibition [174].

On the other hand, studies show that supplementation with high doses of VC may impact changes in the microbiome’s composition. Research conducted by Otten et al. [175] indicated that, in the case that patients were supplemented with VC in doses of 1000 mg, decreases in the relative number of *Lachnospiraceae*, *Bacteroidetes*, *Enterococcus*, and *Gemmiger formicilis* were observed [175]. The latest information about COVID-19 patients shows that the virus is present in gut cells and can affect their condition [176]. This imbalance can also affect the relation of the gut microbiome’s regulating role with the absorption of ascorbic acid. 

### 4.2. High-Dose Intravenous VC Administration

The intravenous usage of antioxidants in severe cases of infection is due to their ability to inhibit cytokine storms [177]. VC has been proven in animal and tissue models to modulate the immunological system by enhancing interferon production, suppressing oxidative stress and thrombosis, inhibiting cytokine storms, and lessening alveolar damage, and it has many other effects on the human body [178]. It is also known to enhance the maturation and proliferation of lymphocytes. VC also has a proven suppressive impact on the cellular expression of ACE2. The cellular expression is the crucial receptor for the SARS-CoV-2 virus to enter the human cells. Thus, VC may hinder the infection process [170]. Based on their research results and available references, Patterson et al. [179] suggested that, in SARS-CoV-2 infected patients, SVCT1 and SVCT2 AA transporters are downregulated, which might be an essential factor related to the severity of COVID-19.

The hypothesis regarding the benefits related to VC administration in severe cases of infection is based on the observation that, in this group of patients, levels of ascorbic acid during the recovery period are very low and, in many cases, undetectable [180,181]. In healthy people, ascorbic acid serum levels are about 0.4–2.0 mg/dL [180]. There is evidence that the majority of critically ill COVID-19 patients has low-to-undetectable VC levels. Tomasa-Irriguible and Bielsa-Berrocal [180] found that, in 82% of patients, within 24 h of ICU admission, the VC was below the normal range, with a mean value of 0.14 mg/dL (+/− SD 0.05 mg/dL). Similar results were observed by Chiscano-Camón et al. [181]—undetectable ascorbic acid levels were found in 94.4% of ICU patients with SARS-CoV-2-associated ARDS. Intravenous AA administration could lead to as much as a 100-fold increase in this vitamin level in the serum within 24 h [182].

Zhao B. et al. [183] found a correlation between patients suffering from multiple organ failure and low serum VC levels. Based on observations conducted during the pandemic, they deducted that high-dose intravenous VC (HDIVC) may prevent disease aggravation in moderate-type COVID-19 and serve as a rescue therapy for severe and/or critical cases of the disease, and they presented a flowchart of a HDIVC protocol proposed by the Shanghai COVID-19 Clinical Treatment Expert Group. The protocol differentiates the severity of the disease. However, it suggests relatively high doses for long-term periods (≥100 mg/kg for 7 days). In 2021, Zhao et al. [183] included SARS-CoV-2-infected patients in the study. They conducted 110 observations before and after COVID-19 therapy, over the course of 7 days. The researchers had two groups of patients: HDIVC-treated patients (*n* = 55) and the control group (*n* = 55). They observed differences in both of the examined groups of patients in forms of infection which worsened from moderate to severe (HDIVC: 7.3%; control: 21.8%; *p* = 0.03). In the HDIVC group, the patients exhibited shorter durations of systemic inflammatory response syndrome (SIRS) (*p* = 0.0004) during the first week and lower SIRS occurrence (9.5% vs. 45.5%, *p* = 0.0086) on day 7 (6–7 days after admission) in comparison to the control group. Additionally, C-reactive protein serum concentrations were lower on the seventh day of observation in HDIVC patients than in the control group [184]. The relationship between VC and CRP in healthy people who have been exposed to some inflammatory factors is known [185,186], but the responsible mechanisms are still being examined.

The results from a retrospective cohort study [187] confirmed the information mentioned above, that HDIVC significantly lowers the levels of serum inflammatory markers, such as hs-CRP, IL-6, and TNF-α. (High-dose VC was intravenously administered over a 6-day course in dosages in excess of 100 mg/kg of body weight. VC was diluted in 50 mL of saline solution and infused within 30 min every 6 h on day 1, and 100 mg VC/kg of body weight was diluted in 50 mL of saline solution and infused within 30 min every 12 h for the next 5 d.) The researchers stated that hyperinflammation is linked to the severity of COVID-19 disease. The patients received over 2 g of VC per day for 6 days. In another study, Xia G et al. [188] found that, besides the lower levels of inflammatory markers after HDIVC (100 mg/kg every 6 h for 1 day, followed by 100 mg/kg every 12 h for an additional 5 days during hospitalization), the positive influence on myocardial damage in consecutively severe and critically ill COVID-19 patients was highlighted [188]. After 21 days of hospital therapy, levels of inflammatory markers (CRP, IL-6, IL-8, and TNF-α) significantly decreased in HDIVC patients.

In addition, during HDIVC therapy, the signs and symptoms in COVID-19 patients have improved. CT scans conducted by Tehrani et al. [189] in 2020 on a group of 54 patients showed that HDIVC (2 g/6 h for 5 days) might improve the rate of pulmonary involvement in moderate-to-critical cases. In addition, VC therapy improved the oxygen saturation and lowered the respiratory rate [189].

Despite the above-mentioned benefits, a meta-analysis by Rawat et al. [190], based on results from 572 cases, states that there is no significant decrease in the mortality rate, length of stay in the ICU, duration of hospital stay, or invasive mechanical ventilation after HDIVC therapy. The number of included papers was six. Three were also evaluated in a meta-analysis by Kwak et al. [191] In four analyses, VC was administrated via HDIVC; in two, the administration was oral. The results of the meta-analyses of the efficacy of using VC in the treatment of COVID-19 is summarized in Table 3.

Those conclusions were confirmed in a randomized, open-label clinical trial. In Iran, 2 groups of ICU patients (*n* = 60) were examined: an HDIVC case group (1.5 g VC every 6 h for 5 days) and a control group [47]. All patients were treated with lopinavir/ritonavir twice daily, with a single dose of hydroxychloroquine (400 mg) on the first day of hospitalization. On the third day of treatment, the case group had significantly higher peripheral oxygen saturation (SpO_2_: 90.5% vs. 88.0%; *p* = 0.014) levels and lower body temperatures (36.73 vs. 37.24; *p* = 0.001) than the control group, but the median length of stay in the hospital was longer in the case group than in the control group (8.5 and 6.5, respectively, *p* = 0.028). The JamaliMoghadamSiahkali [47] trials found no other benefits from the COVID-19 treatment, including mortality and the length of ICU stay. In this study, all patients were treated with hydroxychloroquine. For this reason, the results of this analysis pertain to a combined therapy, and the findings might be related to the combined effect of the hydroxychloroquine and ascorbic acid, rather than being related to AA alone.

Additionally, in a retrospective study from Wuhan conducted by Zheng et al. [195], there was no improvement observed in the clinical outcome, and no association between HDIVC use (2–4 g/day for 1 to 30 days) and the risk of death in severe cases of COVID-19 [195].

Over the last two years, there have been few randomized control trials (RCTs) considering high doses (over 2 g/day, over 5 to 10 days) of intravenous VC treatment in severe COVID-19 patients (Table 4). A meta-analysis from December 2021 suggests that no significant reduction in mortality rate or length of hospital stay was determined. Kwak et al. [191] observed an insignificant reduction in the in-hospital mortality rate among HDIVC patients as compared with the control group. There was also a lower occurrence of SIRS in this group of patients. The final results and the comparison of the effects of VC administration found in RCTs do not support the assumption that VC plays an essential role in treating COVID-19 patients, with the exception of the HDIVC treatment proposed by different authors who treated patients with high doses of VC. However, reports from studies on the long-term results of HDIVC treatment have included the reduction of inflammatory agents in COVID-19 patients [183,184,187,188], limiting the risk of thrombosis and inflammatory conditions affecting the functioning of multiple organs.

### 4.3. Ascorbic Acid Orally Supplementation in COVID-19

There is evidence from randomized clinical trials that VC uptake may shorten the duration of SARS-CoV-2 infection. Hemilä et al. [202] reanalyzed the results obtained by Thomas et al. [196]. They found that ascorbic acid (8000 mg divided over 2–3 times per day, with meals) increased the recovery rate by 70% (95% CI 6.8% to 170%, *p* = 0.025) in ambulatory SARS-CoV-2 infected patients.

A RCT performed by Majidi et al. [201] in December 2021 found that the ICU study group, taking 500 mg of enteral VC per day over 14 days, had a statistically significant higher survival rate. Additionally, the survival duration of patients positively correlated with the time of supplementation [201]. Scientists have also stated that a daily dose of greater than 500 mg drastically lowers its bioavailability in healthy persons. A retrospective clinical trial of 739 critically ill patients hospitalized with UTIs at a governmental tertiary hospital in Saudi Arabia found that low oral doses (1000 mg per day) of VC supplementation do not reduce mortality [201]. Al Sulaiman et al. [203] found that low amounts of VC in a study minimize the incidence of thrombosis in patients, which is the standard of care after COVID-19 infection. This might be related to the decrease in cytokine storms during VC therapy. This effect was observed by Abulmeat et al. [204]. They found that oral supplementation of antioxidants reduces cytokine storm characteristics in an early phase of SARS-CoV-2 infection. The reduction of thrombosis might be associated with the same pathway as found in cardiovascular diseases. Zhu et al. [205] concluded that the protective role of VC in cardiovascular diseases is related to its anti-inflammatory properties. They suspected that the most enriched pathways of VC are JAK-STAT, PD-1, EGFR, FoxO, and the chemokine-signaling pathways.

Vitamin supplementation during care after ICU discharge might be essential [205]. After COVID-19, patients are malnourished, and most experience weight loss (about 25%, or more than 8 kg). Patients are also burdened with psychological disorders, such as depression or anxiety. VC, among other micro- and macroelements, may help them recover faster by diminishing the malnutrition and the scarcity of vitamins and microelements [206].

All of the mentioned reports share one problem concerning the inequality of VC administration programs for patients (see Table 4), limiting the possibility of comparisons between them. The routes of supplementation and the frequency of doses are constantly changing, making comparisons very difficult. In the early stages of COVID-19, the oral administration of 500–1000 mg of VC does not decrease mortality, but it can minimize the risk of thrombosis and the chance of the cytokine storm. Definitively, the administration of VC does not affect the level of mortality. It is possible that, in future, we will know more about the role of VC in avoiding complications, such as the risk of thrombosis after severe COVID-19.

### 4.4. Ascorbic Acid Supplementation in Combined Pharmacological COVID-19 Treatment 

There have been studies on the possible use of VC in combination with other medications in patients with COVID-19. Multiple doses of VC (1.5 g, 4 times per day) were used in the research conducted with hydrocortisone and thiamine, reducing organ failure and mortality from 40.4% to 8.5%. It has also been proven that in a critically ill COVID-19 patient, intravenous sodium ascorbate (60 g) restored arterial pressure, improved renal function, and increased arterial blood oxygen levels. Those findings suggest that megadoses of VC should be trialed as a treatment for sepsis and COVID-19 [207]. It has been shown that their combination can prevent acute kidney damage and reduce mortality in patients with septic shock or sepsis [208]. There is evidence that VC and quercetin co-administration exert synergistic, antiviral action due to overlapping antiviral and immunomodulatory properties. It has been noted that ascorbate’s ability to recycle quercetin increases its efficacy. Quercetin displays a broad range of antiviral properties, for example, virus entry and replication. The co-administration of VC can augment these therapeutic effects. Quercetin and VC may disrupt virus entry and replication, concurrently fortifying the immune response, promoting early IFN production, modulating interleukins, promoting T-cell maturation, and promoting phagocytic activity. This therapeutic option was proposed in addition to the promising treatment options, such as Remdesivir or convalescent plasma [39], for COVID-19 patients. Other combinations which include VC have also been studied.

For example, preliminary results of a clinical trial showed that the treatment of severe COVID-19 with a mixture of MB (methylene blue), VC, and N-acetyl Cysteine is safe and feasible. The study used methylene blue (1 mg/kg) with VC (1500 mg/kg) and N-acetyl Cysteine (1500 mg/kg) orally or intravenously in ICU COVID-19 patients. The immediate effect was to increase the SPO_2_% by reducing met-Hb. (All patients had been receiving 100% oxygen). The delayed effects were the acceleration of the typically slow NADPH–methemoglobin reductase, the improvement of inflammatory marker levels (CRP and LDH), and the decrease in disease severity, which may also have been caused by antimicrobial effects [209]. Other studies suggest the addition of thiamine to a corticosteroid (methylprednisolone), VC, and the anticoagulant, heparin (enoxaparin). The proposed dosing is as follows: methylprednisolone 80 mg loading dose, followed by 40 mg q 12 hourly for at least 7 days, and in patients with an increasing CRP or a worsening clinical status, increase the dose to 80 mg q 12 hourly (and then 120 mg if required), AA 3 g IV q 6 hourly for at least 7 days, thiamine 200 mg IV q 12 hourly, heparin depending on contraindications and whether the patient is at risk, and supplemental oxygen ox. This treatment acts synergistically to restore microvascular function in patients with COVID-19. It has been suggested that the following supplements should also be administered: melatonin (6–12 mg at night), famotidine (40 mg) daily (20 mg in case of renal impairment), vitamin D (2000–4000 u PO daily), elemental zinc (50–75 mg daily), magnesium, atorvastatin (80 mg/daily), and thiamine, which is essential for cellular energy production. Thiamine, along with AA and glucocorticoids, has been shown to reduce delirium in critically ill patients with COVID-19 [210]. Another study hypothesized that combining VC and glycyrrhizic acid is a potential option for treating COVID-19 patients. VC and glycyrrhizic acid individually have potent pharmacological efficacy against viral pneumonia. The research used a strategy of network pharmacology and bioinformatics (including GO and KEGG) to uncover the integrative pharmacological mechanism of VC and glycyrrhizic acid against COVID-19. Findings suggest that VC and glycyrrhizic acid may be able to suppress COVID-19 through their combined antioxidative, antiviral, and anti-inflammatory effects, along with activating the immune system, specifically, T-cell activation and leukocyte adhesion.

Moreover, according to KEGG pathway analysis, the pharmacological mechanisms of VC and glycyrrhizic acid against COVID-19 involve specific modulations of immune responses [211]. Findings suggest that a combination of VC, curcumin, and glycyrrhizic acid may help regulate the immune response to combat COVID-19 infections and inhibit excessive inflammatory responses to prevent the onset of the cytokine storm [212]. Based on recent advanced research, a novel combination of VC, curcumin, and glycyrrhizic acid, which has potential against COVID-19 infection, has been developed. Specifically, it has the potential to regulate the innate immune response by acting on the NOD-like and Toll-like signaling pathways. They promote interferon production and activate and balance T cells. They also regulate the inflammatory response by inhibiting the PI3K/AKT, NF-κB, and MAPK signaling pathways.

The combination of VC and other widely used agents with antioxidant properties, i.e., quercetin, methylene blue, or thiamine, results in increased efficacy through additive effect. The following advantages have been noted: an increased saturation rate, renal function and vascular barrier improvement, and an increased anti-inflammatory effect, requisite to lipid and carbon metabolism regulation. In COVID-19 prevention and/or in mild cases of infection, vitamin C supplementation possibly benefits patients.

## 5. Conclusions

The facts that the polymorphism of solute carrier family genes affects the uptake of VC according to the phylogenic origin of patients; that it affects their health conditions, such as T2DM, which increases the risk of hospitalization and death in COVID-19; and that there are interactions between the solute carrier family and a broad spectrum of cardiological diseases all suggest an increased susceptibility of patients to complications and risk of death.

Surprisingly, despite the best efforts, only a few publications have focused directly on the role of VC transporters and their modulation as factors related to the severity of COVID-19. Although there are many publications concerning the role of VC in treating patients with COVID-19, no paper has simultaneously considered the nuances of VC uptake, transport, and genetic variability. The genetic variability related to SNPs is significant in differentiating populations of people across the world. The potential role of SNPs in the susceptibility to a spectrum of diseases with differing severities seems to be well-known, and, according to collected data, the comparison between allele frequency, an abundance of bacteria species in gut microbiota, and a correlation with the plasma levels of VC should be developed as soon as possible, especially since indirect sources indicate that, for example, the abundance of *E. coli* in the gut microbiota of patients with severe symptoms of COVID-19 is higher than in the gut microbiota of healthy patients.

Undoubtedly, studies of VC’s influence on the prevention of infection and treatment during COVID-19 should include an analysis of the *slc23* and *slc2* family of genes’ polymorphism and expression as the essential factors affecting the uptake and regulation of ascorbic acid levels in organisms. Additionally, studies devoted to research on the gut microbiome could affect the conclusions reached by analyzing glycyrrhizic acid, VC, and curcumin. These compounds affect gut microbiota and can suppress or induce the expression of many genes associated with the regulation of the cell cycle, apoptosis, cell adhesion, phosphatases, and kinases, but they also act as epigenetic modulators.

On the other hand, there are many mutually cancelling publications concerning the role of VC in minimizing the symptoms of COVID-19. The influence of VC could be cleared up if it is related to genetic background. Undoubtedly, the role of VC in human organisms has been efficiently clarified, but many unexplored areas still require intensive basic and clinical research. The most puzzling phenomena are the broad spectrum of VC’s activities and the equally wide spectrum of results obtained with different modes of administration and different dosages. Finally, knowing that VC is a most-welcomed supplement in the human organism, and even above-average dosages of that vitamin are not so prone to risk, it could be confirmed that even rare cases of a positive impact could be considered as positive evidence of a supportive effect for VC.

The positive influence of VC has been mentioned as improving the immune system [101], regulating the immune system [121,122,123,124,125], decreasing ROS [35,125,144,149], and reducing inflammatory markers [38,43]. The positive influence of VC has been described in the apoptosis of cancer tumors [116,119]. In viral infections, including COVID-19, the positive effect of VC administration has also been reported [135,136,137,138,203,209]. The neutral effect of VC has been mentioned in the case of patients with COVID-19 in a few publications [47,48,190,191,192,193,194,196,198,199,200], and the adverse effects of the administration of high doses of VC have been mentioned as an aspect of urinary and kidney stones [88,89,90] and nephropathy [45]. These last effects were found to be neutral. The negatives mainly concern problems with the continuous and extended administration of the vitamin, rather than being basically adverse effects. In light of aforementioned information, it can be concluded that the balance between the positive and negative effects should be considered only when there is evidence of antagonism between vitamin C and other therapeutic agents. VC does not harm, so using that vitamin as an additive to the primary treatment will not be wrong because there is always a chance that HDIVC will improve the effects.

This leads to the conclusion that VC helps, rather than harms, but it should not be considered a supplement that plays an essential role in COVID-19 treatment.

## Figures and Tables

**Table 1:** Effect of VC treatment/supplementation among COVID-19 patients.

Study Design	Patients	Dose of VC	Results	References
Case report	A 74-year-old woman	1 g twice a day (for a total of 10 days)	fewer days on mechanical ventilation; shorter ICU stay; earlier recovery	[42]
Case series	17 patients	1 g every 8 h for 3 days	reduced mortality; reduced number of intubations; decreased inflammatory markers (ferritin and D-dimer); reduced need for FiO_2_	[38]
Retrospective case series	12 patients	A median of 162.7 (71.1–328.6) mg/kg (body weight)/day in severe patients, and 178.6 (133.3–350.6) mg/kg/day in critical patients	significant decrease in C-reactive protein, lymphocyte, and CD4+ T-cell counts; improvement of PaO_2_/FiO_2_; improvement of SOFA score	[43]
Case report	A 29-year-old man	VC treatment together with inhalation therapy	the patient died of tension pneumothorax	[44]
Case report	2 patients (a 50-year-old and a 71-year-old man)	200 mg/kg/day, for 96 h	an adverse effect associated with high-dose intravenous VC administration; ATI and oxalate nephropathy are likely caused by excessive VC	[45]
Retrospective study	102 patients	73 patients received supplementation with VC and zinc	high mortality no change in overall survival of patients	[46]
Open-label, randomized, and controlled trial	30 patients with severe COVID-19 infection	1.5 g of IV VC every 6 h for 5 days plus lopinavir/ritonavir and oral hydroxychloroquine vs. lopinavir/ritonavir and oral hydroxychloroquine alone	no difference in the length of ICU stay, intubation rate, or mortality rate	[47]
Open-label, randomized, and controlled trial	75 patients	50 g/kg/day	no significant difference was found in the need for mechanical ventilation or mortality	[48]

ICU—intensive care unit; SOFA—Sequential Organ Failure Assessment score; ATI—acute tubular injury; COVID-19—coronavirus disease 2019.

**Table 2:** The role of selected antioxidants in the course of COVID-19.

Antioxidant	Properties	Role in COVID-19	References
Vitamin C	a cofactor of many enzymes; protects neutrophils and phagocytes from the damage that occurs after oxidative burst; modulation of nuclear transcription factor kappa B (NFkB); attenuation of pro-inflammatory cytokine production	inhibits the production of superoxide (O^2−^) and peroxynitrite (ONOO^–^) by inhibiting O^2−^ NADPH oxidase production and mRNA expression induced nitric oxide synthase (iNOS); in patients with septic shock, decreases MOF, SOFA score, ICU stay time, oxidative stress (OS), and inflammation	[54,55]
Vitamin D	reduces the expression levels of proinflammatory type 1 cytokines, such as IL-12, IL-16, IL-8, TNF-α, and IFN-γ, while increasing type 2 cytokines, such as IL-4, IL-5, IL-10, and regulatory T cells	a potential role in the suppression of the cytokine storm during COVID-19	[56,57]
Vitamin E	the lipophilic antioxidant in cell membranes; scavenger of O^2−^ and hydroxyl (OH) radicals	improvement of the immune response an antioxidant in the acute phase of COVID-19 in patients with septic shock, decreases MOF, SOFA score, ICU stay time, oxidative stress (OS), and inflammation	[58]
Melatonin (MT)	sequesters ROS, thus protecting lipids in cell membranes, proteins in the cytosol, DNA, and mitochondria; stimulates antioxidant enzymes, such as catalase (CAT), superoxide dismutase (SOD) isoforms, GPx, and GR	improves sleep habits, reduces anxiety, and boosts immunity; in patients with septic shock, decreases MOF, SOFA score, ICU stay time, oxidative stress (OS), and inflammation decrease; can alleviate septic shock via the NLRP3 pathway; prevents sepsis-induced kidney damage, septic cardiomyopathy, and liver injury	[59,60,61]
Pentoxifylline (Px)	antioxidant and anti-inflammatory effects, such as maintaining GSH levels and mitochondrial viability; inhibition of TNF-α production and maintenance of vascular endothelial function	in patients with septic shock, decreases MOF, SOFA score, ICU stay time, oxidative stress (OS), and inflammation	[33,35]
N-acetylocysteine (NAC)	reduces the incidence of pneumonia	binds to Cys-145, the active site of the M protein, and therefore inhibits protease activity and viral replication; a potentially specific, first-line drug for SARS-CoV-2; can help to slow down the aggressive and lethal development of COVID-19 with the use of a moderate dose	[62,63]
Zinc	regulates inflammatory activity; antiviral and antioxidant functions	increases the number of cytotoxic T lymphocytes; reduces the number of activated T helper cells; improves cellular immunity, reduces oxidative stress and the production of chronic inflammatory cytokines	[64]

MOF—multi-organ failure; SOFA—Sequential Organ Failure Assessment; ICU—intensive care unit; OS—oxidative stress; SARS-CoV-2—severe acute respiratory syndrome coronavirus 2; COVID-19—coronavirus disease 2019.

**Table 3:** Meta-analyses conducted on the topic of VC use in COVID-19 patients.

Reference	Month, Year	No. of Studies	PICO	PRISMA	RCTs Only	Number of Patients	VC Dose	Adverse Effects	Conclusion
[190]	November–December 2021	6	YES	YES	YES	572	≥1 g daily, IV or oral	ND	No significant benefit
[192]	February 2022	7	NO	YES	NO (3 RCT, 4 retrospective studies)	807	≥2 g daily HDIVC	ND	No significance in mortality rate or disease severity
[193]	January 2022, Beran A. et al.	9	YES	YES	NO (4 RCT, 5 retrospective studies)	1488	≥1 g daily, IV or oral	ND	No significance in mortality, intubation rate, or hospitalization length
[191]	March 2022	5	YES	YES	NO (3 RCT, 2 retrospective studies)	374	≥2 g daily HDIVC	No significance	No significance in mortality rate or hospitalization length
[194]	February 2022	11	NO	YES	NO (4 RCT, 7 retrospective studies)	1807	≥1 g daily, IV or oral	ND	No significance in mortality, a longer length of stay for VC group

Abbreviations: IV—intravenous; HDIVC—high-dose intravenous vitamin C; ND—no data.

**Table 4:** RCTs conducted on the topic of VC use in COVID-19 patients.

Reference	Study Design	No. of Patients	VC Age	VC Sex, Female (%)	C Age	C Sex	Patient	Disease Severity	Usage of VC	Conclusion
[48]	RCT	150	52 ± 11	ND	53 ± 12	ND	Inpatients	Severe	50 mg/kg/day IV	No impact on mortality or need for mechanical ventilation; difference in length of hospitalization
[196]	RCT	214	46 ± 15	33(69)	42 ± 15	31 (62)	Outpatients	Mild/moderate	8000 mg/day OR	No significant decrease in the duration of symptoms compared with standard of care
[47]	RCT	60	58 ± 18	15(50)	61 ± 16	15 (50)	Inpatients	Severe	4500 mg/day IV	No significantly better outcomes in the IV VC group
[197]	RCT	56	66 ± 11	12(44.4)	67 ± 14	7 (24)	Inpatients	Severe	24,000 mg/day IV	No significant difference in invasive mechanical ventilation-free days/28 days between groups
[198]	Pilot randomized trial	20	59 ± 19 7(35)				Inpatients	Severe	6000 mg/day IV	No considerable improvement in the clinical status of patients
[199]	RCT	72	36 ± ND	14(37)	37 ± ND	12 (35)	Inpatients	Moderate (VAP excluded)	1000 mg/day OR	Vitamin C and vitamin E failed as an adjunctive treatment for COVID-19 patients
[200]	RCT	60	51 ± 17	15(50)	53 ± 7	14 (47)	Inpatients	Severe	2000 mg/day ND	No significant difference in length of hospitalization
[201]	RCT	120	59 ± 15	12(39)	64 ± 16	28 (42)	Inpatients	Severe	500 mg/day OR	Higher mean survival duration compared to control group

RCT—Randomized controlled trial; VC Age—age of people taking vitamin C; VC Sex—sex of people taking vitamin C; C Age—age of people in the control group not taking vitamin C; C Sex—sex of people in the control group not taking vitamin C; VC—vitamin C; ND—no data; VAP—ventilator-associated pneumonia; IV—intravenous; OR—oral; COVID-19 coronavirus disease 2019.

## Data Availability

Not applicable.
